# Tumor necrosis factor receptor 1 (TNFRI) for ventilator-associated pneumonia diagnosis by cytokine multiplex analysis

**DOI:** 10.1186/s40635-015-0062-1

**Published:** 2015-09-16

**Authors:** Ignacio Martin-Loeches, Lieuwe D. Bos, Pedro Povoa, Paula Ramirez, Marcus J. Schultz, Antoni Torres, Antonio Artigas

**Affiliations:** Multidisciplinary Intensive Care Research Organization (MICRO), Department of Clinical Medicine, Trinity Centre for Health Sciences, St James’s University Hospital, James’s Street, Dublin 8, Ireland; CIBER enfermedades respiratorias (CIBERES), Critical Care Center, Sabadell Hospital, Corporación Sanitaria Universitaria Parc Taulí, Universitat Autonoma de Barcelona, Sabadell, Spain; Department of Intensive Care and Laboratory of Experimental Intensive Care and Anesthesiology, Academic Medical Center, University of Amsterdam, Amsterdam, The Netherlands; Polyvalent Intensive Care Unit, São Francisco Xavier Hospital, Centro Hospitalar de Lisboa Ocidental, Lisbon, Portugal; Nova Medical School, CEDOC, New University of Lisbon, Lisbon, Portugal; CIBER enfermedades respiratorias (CIBERES), Respiratory Disease Department, Hospital Clínic i Provincial de Barcelona, IDIBAPS, Barcelona, Spain; Intensive Care Unit, University Hospital La Fe, Valencia, Spain

## Abstract

**Background:**

The diagnosis of ventilator-associated pneumonia (VAP) is challenging. An important aspect to improve outcome is early recognition of VAP and the initiation of the appropriate empirical treatment. We hypothesized that biological markers in plasma can rule out VAP at the moment of clinical suspicion and could rule in VAP before the diagnosis can be made clinically.

**Methods:**

In this prospective study, patients with VAP (*n* = 24, microbiology confirmed) were compared to controls (*n* = 19) with a similar duration of mechanical ventilation. Blood samples from the day of VAP diagnosis and 1 and 3 days before were analyzed with a multiplex array for markers of inflammation, coagulation, and apoptosis. The best biomarker combination was selected and the diagnostic accuracy was given by the area under the receiver operating characteristic curve (ROC-AUC).

**Results:**

TNF-receptor 1 (TNFRI) and granulocyte colony-stimulating factor (GCSF) were selected as optimal biomarkers at the day of VAP diagnosis, which resulted in a ROC-AUC of 0.96, with excellent sensitivity. Three days before the diagnosis TNFRI and plasminogen activator inhibitor-1 (PAI-1) levels in plasma predicted VAP with a ROC-AUC of 0.79. The *slope* of IL-10 and PAI-1 resulted in a ROC-AUC of 0.77. These biomarkers improved the classification of the clinical pulmonary infection score when combined.

**Conclusions:**

Concentration of TNFRI and PAI-1 and the slope of PAI-1 and IL-10 may be used to predict the development of VAP as early as 3 days before the diagnosis made clinically. TNFRI and GCSF may be used to exclude VAP at the moment of clinical suspicion. Especially TNFRI seems to be a promising marker for the prediction and diagnosis of VAP.

**Electronic supplementary material:**

The online version of this article (doi:10.1186/s40635-015-0062-1) contains supplementary material, which is available to authorized users.

## Background

Ventilator-associated pneumonia (VAP) is a frequently encountered infection in mechanically ventilated patients (MV). While the incidence of VAP has been decreasing over the last years, due to several preventive strategies [1], the burden of this complication remains high with significant morbidity and mortality. While the diagnosis of VAP is a challenge [2, 3], the most important aspect to improve outcome is early recognition and the initiation of the appropriate empirical treatment [4]. Too late initiation or inappropriate antibiotic therapy is associated with adverse outcome [5].

The ideal biological marker for VAP would allow for a rapid diagnosis, have a prognostic value, and facilitate therapeutic decision-making [6]. So far, only C-reactive protein (CRP) and procalcitonin (PCT) were found to fulfill some of these properties [7]. CRP, however, lacks specificity and often rises when VAP is already ongoing [8]. While use of PCT was shown to reduce of duration and to prevent unnecessary start of antibiotic therapy, alike CRP, it has no value in the early recognition of VAP [9].

We hypothesized that biological markers in plasma, other than CRP and PCT, can add to the diagnostic process of VAP in two distinct ways; first, they could rule out VAP at the moment of clinical suspicion; second, they could rule in VAP before the diagnosis can be made clinically. To test this hypothesis, we performed an unbiased search for plasma biological markers in a cohort of patients at high risk of developing VAP, using cytokine multiplex analysis. We specifically investigated the diagnostic accuracy of biological markers in the 3 days preceding and at the moment of VAP diagnosis.

## Methods

### Study design, ethical approval, and informed consent

The Biomarkers in the diagnosis and management of Ventilator-Associated Pneumonia (BioVAP) study was a prospective, multicenter, observational study in 4 university teaching hospitals. The institutional review boards of the participating hospitals reviewed and approved the study design. BioVAP was registered at www.clinicaltrials.gov. The protocol was approved by the local ethics committee of the four hospitals. Research that is reported in the manuscript is in compliance with the Helsinki Declaration. (NCT02078999).

### Study subjects

All patients admitted to the participating ICU were screened for inclusion. For each patient, only the first ICU admission and the first VAP episode were included in the study. All patients admitted to one of the participating ICUs were screened for inclusion if they were mechanically ventilated for >72 h, which included any patient without evidence of pneumonia in the chest x-ray, that was not receiving antibiotics for at least 5 days before ICU admission, with an expected length of mechanical ventilation >72 h and in whom antibiotics were not prescribed on admission by the attending physician (the use of antibiotics as prophylaxis was not an exclusion criteria).

### Inclusion and exclusion criteria

All patients admitted to one of the participating ICUs were screened for inclusion if they were mechanically ventilated for >72 h, which included any patient without evidence of pneumonia in the chest x-ray, that was not receiving antibiotics for at least 5 days before ICU admission, with an expected length of mechanical ventilation >72 h and in whom antibiotics were not prescribed on admission by the attending physician. Exclusion criteria included current and past participation in another intervention trial conflicting with the present study, previous endotracheal intubation longer than 12 h during the previous 30 days, patients with documented bronchiectasis, cystic fibrosis, witnessed pulmonary aspiration either prior or at intubation, BMI > 40, age below 18 years, absence of consent, and pregnancy.

### Primary endpoint and secondary endpoints

The primary endpoint was the best combination of biomarkers for accurate diagnosis of VAP. Secondary endpoints were calibration and net classification improvement when the biomarkers were combined with the clinical pulmonary infection score [10].

### VAP definition

Definition of pneumonia, microbiologic processing and antimicrobial treatment, and clinical diagnosis of ICU-AP was based on either (a) clinical criteria (new or progressive radiological pulmonary infiltrate together with at least two of the following: temperature >38 °C or <36 °C), leukocytosis >13,000/mm^3^ or leukopenia <4000/mm^3^, or purulent respiratory secretion; or (b) a simplified clinical pulmonary infectious score (CPIS) ≥6 points (after 48 h of ICU admission and invasive mechanical ventilation) [11]. VAP was defined as a pulmonary infection arising more than 48 h after tracheal intubation, with no evidence of pneumonia at the time of intubation or the diagnosis of a new pulmonary infection if the initial admission to ICU was due to pneumonia. Microbiologic confirmation of pneumonia was defined by the presence of at least 1 potentially pathogenic microorganism (PPM) in respiratory samples above predefined thresholds (for bronchoalveolar lavage specimens, >104 CFU/mL; for sputum or tracheobronchial aspirate specimens, 105 CFU/mL). Microbial identification and susceptibility testing are detailed elsewhere [12]. Ventilator-associated tracheobronchitis episodes were not included in the study. The presence or absence of a new or progressive radiological pulmonary infiltrate was based on the interpretation of the chest radiograph by board-certified radiologists who were blinded to the study. All classifications, including the radiographs and laboratory data used in their determinations, were prospectively reviewed by one of the investigators (IM-L) and confirmed by a second investigator (PP).

### Follow-up

Patients were followed up till the 21st day, the day of successful weaning and extubation, the day of a non-VAP infection, or the day of clinical diagnosis of VAP, whatever came first (for definitions, see ESM file). Additionally, death or ICU discharge as well as hospital discharge was assessed. At 90th day, a telephonic interview was performed for outcome assessment.

### Luminex

In brief, 18 biomarkers were measured per sample (see list of abbreviations at the end of the manuscript and the online supplement for an overview). Cytokine detection using multiplex bead array assays exhibits high degrees of intra-assay (<10 % variation) and inter-assay (10–20 % variation) precision [13, 14]. Cytokine detection by Luminex xMAP technology is comparable to that with enzyme-linked immunosorbent assay (ELISA; correlation coefficient *r* ranges from 0.75 to 0.99) [13–15].

### Power calculation

In this study, we aimed to identify very sensitive diagnostic markers of VAP. With an anticipated sensitivity of 95 % and minimal acceptable lower confidence limit of 65 % a sample size of minimally 16 patients per group was needed [16].

### Statistical analysis

Differences between the groups were compared using the Mann–Whitney U test for continuous variables and Fisher exact for categorical variables. Data were summarized using the median and inter-quartile range for continuous variables and with count and percentage for categorical variables. All analyses were performed in R statistics using R studio [17]. *p* values below 0.05 were considered significant.

First, individual biological markers were compared cross-sectionally between patients that developed VAP (at the moment of diagnosis) and did not develop VAP (at a similar moment) using the Mann-Whitney U test. That analysis was repeated for the data from 3 days before the diagnosis. Second, the absolute change in biomarker concentration was calculated per patient. These slopes were compared between VAP and no VAP using the Mann-Whitney U test. Third, the area under the receiver operating characteristic curve (ROC-AUC), optimal cut-off, sensitivity, specificity, and likelihood ratios were calculated per biomarker and biomarker slope. Fourth, the best biomarker combination was investigated. All biomarkers and biomarker slopes, respectively, that had an ROC-AUC above 0.7 qualified for inclusion in the initial logistic regression model. Backward selection based on the Akaike Information Criteria was performed with the rms package [18]. Receiver operating characteristics were calculated using the pROC package [19] and calibration was visualized with using ggplot2 [20]. The group label (VAP yes/no) was permutated for 1000 times to calculate the probability that a similar or better discrimination value would be found based on chance. Diagnostic accuracy was compared between the biomarker models and the CPIS and the slope of the CPIS. Finally, the net-reclassification index and the integrated discrimination improvement were calculated for the combination of CPIS (slope) and biomarker (slope) [10].

## Results

### Patients

**Fig. 1:**
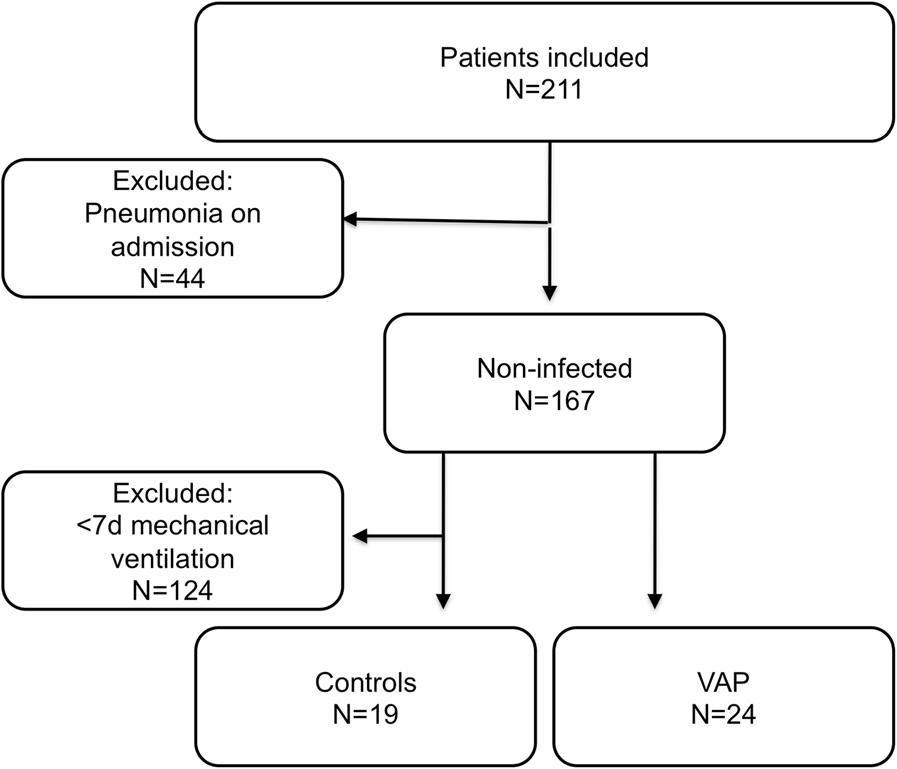
Patient flow

We collected data and samples from 43 patients (Table 1). Twenty-four patients developed microbiology confirmed VAP and 19 patients were considered controls (Fig. 1). The SAPS II and SOFA score on admission was higher in patients who developed VAP. Patients who developed VAP had a higher mortality rate and longer stay in ICU compared to control patients.

**Table 1:** Patient characteristics

Variable	Units	Control		VAP		*p* value
		*N* = 19		*N* = 24		
Age	Mean SD	49.89	23.51	53.96	16.79	0.53
Male	N %	10	52.6	18	75	0.21
CODP	N %	0	0	4	16.7	0.12
Diabetes	N %	2	10.5	2	8.3	1.0
Immunosuppression	N %	0	0	1	4.2	1.0
Hearth failure	N %	2	10.5	1	4.2	0.58
Liver failure	N %	0	0	1	4.2	1.0
Renal failure	N %	1	5.3	2	8.3	1.0
Apache II	Median IQR	22	[17.5–24.5]	25.5	[20.75–33]	0.11
SAPS II	Median IQR	44	[23.5–50.5]	57	[46.75–71]	0.004
SOFA	Median IQR	6	[5–8]	8	[6–11]	0.04
WBC	Median IQR	10.4	[8.3–14.27]	14.5	[9.59–16]	0.15
CPIS	Median IQR	3	[1–4]	3	[1–3.5]	0.59
ICU-LOS	Median IQR	8	[6–10]	19.5	[14–23.75]	<0.001
Hospital-LOS	Median IQR	21	[15.5–24]	30	[20–43.25]	0.08
Mortality	N %	0	0	10	41.7	0.001

*COPD* chronic obstructive pulmonary disease, *APACHE II* Acute Physiology and Chronic Health Evaluation II, *SAPS* simplified acute physiology score, *WBC* white blood cell count, *CRP* C-reactive protein, *PCT* procalcitonin, *CPIS* clinical pulmonary infection score, *LOS* length of stay

### Serum levels of biomarkers measured by multiplex

Additional file 1: Table S1 shows the values for biomarkers studied in patients with or without VAP at the day of diagnosis. Compared with the non-VAP group, patients in the VAP group had a higher GCSF, IL-10, IL-6, IL1RA, IL-8, TNFa, and TNFRI levels (Additional file 1: Figure S1). The same analysis was also performed 3 days before VAP diagnosis in order to determine predictive accuracy of these plasma biomarkers before clinical symptoms started (Additional file 1: Table S2). TNFRI, PAI-1, IL-8, and IL-12-P40 were significantly different between patients that were to develop VAP and those that were not to develop VAP, 3 days before the diagnosis (Additional file 1: Figure S3). Additional file 1: Table S3 shows the slopes of these biomarkers (log 10 transformed concentrations) in patients with and without VAP. Only the slopes of IL-12 and PAI-1 were significantly different between patients that did and did not develop VAP.

### Best biomarker combination for ruling out VAP

**Fig. 2:**
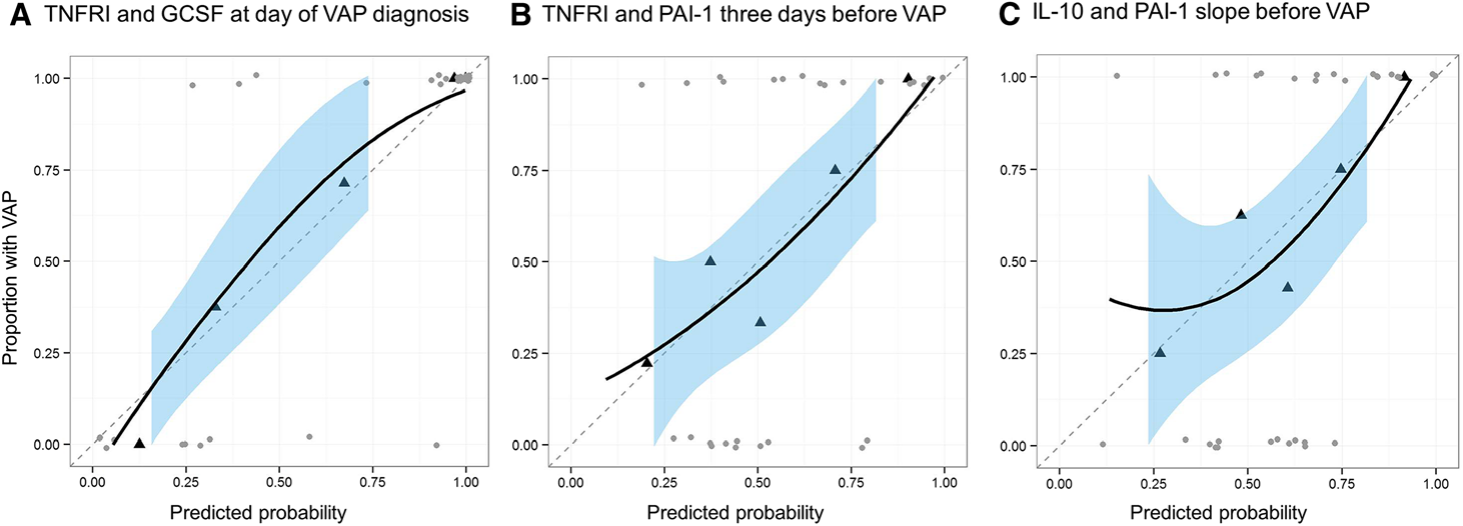
Calibration plots at the three different time points. Calibration plots for the biomarker models. X-axis: predicted probability of VAP by the biomarker concentrations. Y-axis: the proportion of patients that actually had VAP. The *grey dots* show the predicted probabilities of the individual patients. The *black triangles* show the quantile summary and the *black line* the smoothed association between predicted and actual probability of VAP. The *grey dotted line* shows the ideal situation where predicted and actual probability are equal. **a** TNFRI and GCSF at the day of VAP diagnosis. **b** TNFRI and PAI-1 3 days before VAP. **c** IL-10 and PAI-1 slope before the diagnosis of VAP

TNFa, TNFRI, IL-6, IL-8, IL-10, and GCSF were included in the initial logistic regression model. Backward selection kept only TNFRI and GCSF as independent variables (TNFRI (10-log), coefficient = 8.9, *p* = 0.01, GCSF (10-log), coefficient = 2.6, *p* = 0.03) and resulted in excellent discrimination with a ROC-AUC of 0.96 (95 % CI: 0.90–1.0; see Table 2 and Additional file 1: Figure S2). This model achieved a very good sensitivity and specificity (sensitivity: 96 %, specificity: 87 %, LR+: 7.2, LR-: 0.05) and was well calibrated (Fig. 2a). None of the simulated scenarios (0/1000; 0 %) in which the group label (VAP yes/no) was permutated resulted in an equal or greater ROC-AUC.

**Table 2:** Test characteristics of CPIS and biomarkers

Time point	CPIS	Biomarkers	Combined	NRI	IDI
	(ROC-AUC)	(ROC-AUC)	(ROC-AUC)		
Moment of diagnosis	0.94	0.96	1.0	Inf	0.37; *p* = 0.001
Three days before	0.59	0.79	0.74	0.8; *p* = 0.046	0.19; *p* = 0.039
Slope	0.95	0.77	0.97	0.4; *p* = 0.36	0.03; *p* = 0.38

*ROC-AUC* area under the receiver operating characteristic curve, *NRI* net reclassification index, *IDI* integrated discrimination index

### Best biomarker combination for ruling in VAP 3 days before clinical diagnosis

TNFRI and PAI-1 were included after univariate analysis and both were kept in the model after backward selection (TNFRI (10-log), coefficient = 14.8, *p* = 0.03, PAI-1 (10-log), coefficient = 3.6, *p* = 0.15). This model provided moderate to good discrimination with a ROC-AUC of 0.79 (95 % CI: 0.66–0.93; see Table 2 and Additional file 1: Figure S4). This model achieved a moderate sensitivity and specificity (sensitivity: 63 %, specificity: 89 %, LR+: 5.9, LR-: 0.42) and was well calibrated (Fig. 2b). Several simulations (25/1000; 2.5 %) with permutated group labels resulted in an ROC-AUC that was greater or equal to 0.79.

### Best biomarker slope combination for ruling in VAP by slope analysis

TNFa, IL-8, IL-10, IL-12, HSP-8, and PAI-1 slopes were included in the logistic regression model. IL-10 and PAI-1 slopes were kept in the model after backward selection (IL-10 slope, coefficient 6.2 × 10^−3^, *p* = 0.13 and PAI-1 slope, coefficient −1.3 × 10^−5^, *p* = 0.02). The model resulted in good discrimination with a ROC-AUC of 0.77 (95 % CI: 0.62–0.92; see Table 2 and Additional file 1: Figure S5). The *biomarker slope model* achieved a sensitivity of 58 % and specificity of 93 % (LR+: 8.8, LR-: 0.45) and was well calibrated (Fig. 2c).

### Combination with clinical prediction score

The CPIS presented a good performance for diagnosis with a ROC-AUC of 0.94 (95 % CI: 0.86–1.0) at the day of diagnosis but was not discriminatory 3 days before (ROC-AUC: 0.64, 95 % CI: 0.37–0.87). The slope of the CPIS provided excellent discrimination (ROC-AUC: 0.95, 95 % CI: 0.86–1.0). Table 2 shows the combined ROC-AUC of CPIS and the biomarker models described before and the net reclassification improvements (NRIs) and integrated discrimination improvement (IDI); two measures of improvement of classification/discrimination that take the pre-test probability based on a clinical score into account [3].

## Discussion

Diagnosis and prediction of VAP has been widely studied in order to find a single biomarker that can achieve this goal [21–23]. To our knowledge, this is the first study that aimed to evaluate the inflammatory response in a multilateral fashion by the use of a prospective design for prediction and diagnosis of VAP. TNFRI and PAI-1 may be used to predict the development of VAP as early as 3 days before the diagnosis made clinically. Furthermore, the slope of changes in concentrations of IL-10 and PAI-1 also provided accurate information on the development of VAP. Finally, TNFRI and GCSF could be used to exclude VAP at the moment of clinical suspicion. Especially, TNFRI seems to be a promising marker for the prediction and diagnosis of VAP.

The diagnosis of VAP has been a matter of debate for the last years [24]. Whereas biomarkers have been proposed for diagnosis, current guidelines suggest that the use of such might improve diagnosis accuracy but until date their use is not extended [25]. In addition, current guidelines (ATS) do not recognize the use of biomarkers for VAP diagnosis. The current guidelines support a clinical approach for diagnosis but when compared to postmortem studies, sensitivity and specificity can be as low as 69 and 75 %, respectively [26]. This clearly manifests a room for improvement, especially in these days when the molecular medicine is more available and feasible [27]. The use of multiple-marker medicine is closer to the clinical reality in pneumonia diagnosis than more strict criteria usually considered in most clinical studies.

To date, several studies have attempted to test the performance of several biomarkers for VAP diagnosis. The best-studied ones are CRP and PCT. However, some other have also been proposed. Conway Morris et al*.* prospectively studied 72 patients until VAP diagnosis and found that the levels of IL-8 and IL-1β in BALF could be used for discrimination. Conversely, all measured cytokines and inflammatory mediators in serum showed similar concentrations in the VAP and non-VAP groups. No serum marker appeared to have potential value for discriminating VAP from non-VAP, though a trend in this direction was observed for sTREM-1 [28]. Interestingly, the authors used enzyme-linked immunosorbent assay (ELISA) for cytokine measurement. The availability of methods to measure different inflammatory mediators or biomarkers with high sensitivity and specificity is extremely important. ELISA has been the most widely used and best validated method; however, the main limitation is by its ability to measure only a single protein in each sample. Recent developments in serum biomarker quantification technology include multiplex arrays, which benefit from the better evaluation of the complexity and dynamic nature of inflammatory responses, and chemiluminescence technology, which is more sensitive than chromogenic detection in traditional ELISA [29]. In our study, we included a total of 18 biomarkers that comprised inflammation (pro- and anti-inflammatory cytokines), coagulation, apoptosis, and early phases of immunity response with the use of multiplex technology and might represent a better accuracy.

Most studies investigating biological markers in intensive care unit patients have only looked at these predictors cross-sectionally. In other words, only the absolute concentrations were used to identify the most relevant markers. We tried to capture the dynamic properties of the biomarker’s concentration by looking at the slope as a predictor. That approach has been successful before in predicting the resolution of sepsis in patients with community-acquired pneumonia [6] and also provided good diagnostic accuracy in our study. Interestingly, the same two markers that were selected in the cross-sectional analysis 3 days before the diagnosis of VAP were also selected in the “slope analysis” (IL-10 and PAI-1). The observed consistence in selection of these markers increases the likelihood that these markers indeed could be used in the diagnosis of VAP.

Clinical findings are still the most important features for VAP diagnosis. In our study, modified CPIS that combines clinical, radiological, and physiological features presented a good performance for diagnosis alone with an ROC-AUC of 0.98 and when combined with GCSF and TNFRI, the performance reached 1.0.

In our study, we found that the combination of TNFRI and GCSF achieved a very good sensitivity and specificity (sensitivity: 96 %, specificity: 87 % ) at the day of diagnosis of VAP and TNFRI and PAI-1 showed a moderate to good discrimination 3 days before the diagnosis (AUC 0.79). Therefore, to the best of our knowledge, TNFRI seems to be a promising marker not only for diagnosis but also for prediction. Identifying early markers of VAP has been difficult due to the complex nature of this illness and TNFRI provide additional information over clinical variables and add mechanistic insight into VAP. TNFRI is a positive T-cell co-stimulatory molecule important for the timing of cytokine responses [21]. Several theories have been recently revisited with the development of a bad progression of sepsis and the development of recurrent infections in ICU [22]. TNFR1-dependent apoptosis and interleukin-6 induction has been described elsewhere [23]. Production of both proinflammatory and immunosuppressive cytokines is observed from the very first hours following diagnosis of VAP but the most interesting finding is that the presence of TNFRI was determined 3 days before VAP diagnosis.

Our study has several strengths. First, this is a multicenter prospective observational study that limits the potential bias of center selection that allows generalization of its results. This is the first study to assess biomarker dynamics before VAP diagnosis. Second, we have analyzed inflammatory/anti-inflammatory, coagulation, apoptosis, and early phases of immunity response with the use of multiplex technology for VAP diagnosis but also we have incorporated and included in the analysis the clinical component. Third, we have performed our analysis with the use of chemiluminescence technology in order to obtain a better dynamic range, more quantitative results, and better signal stability over time.

Our study, on the other hand, has two important limitations. First, the nonrandomized and observational nature of the study design bears the potential of unmeasured confounders that may have caused differences in therapeutic and supportive approach. However, all the patients included were prospectively followed in order to determine that VAP correctly diagnosed. The presence or absence of a new or progressive radiological pulmonary infiltrate was based on the interpretation of the chest radiograph by board-certified radiologists who were blinded to the study. All classifications, including the radiographs and laboratory data used in their determinations, were prospectively reviewed and confirmed by two investigators. Samples were taken daily but we analyzed those at the time of VAP diagnosis and 3 days before the onset to implement our study design in the current clinical practice. Second, in order to comply with the above-mentioned recommendations, we had to exclude a large number of patients, namely in order to avoid interferences of ongoing antibiotic therapy at the time of initiation of mechanical ventilation. Consequently, the changes overtime in studied biomarkers were only due to the presence or absence of VAP. Therefore because the number of patients included in the study is low and our results need to be validated to minimize a selection bias. It is important to highlight that this is a pilot study and more study subjects or an a priori test of the models in a validation cohort would be needed to confirm our results.

## Conclusions

Therefore, to the best of our knowledge, TNFRI seems to be a promising marker not only for diagnosis but also for prediction. Identifying early markers of VAP has been difficult due to the complex nature of this illness, and TNFRI provides additional information over clinical variables and adds mechanistic insight into VAP. Data should be interpreted cautiously because of the nature of a pilot hypothesis-generating study and an a priori test of the models in a validation cohort would be needed to confirm our results in larger populations.

## Additional file

Additional file 1:
**Electronic supplement material.** (DOC 702 kb)

## Abbreviations

GCSF: granulocyte-colony stimulating factor
IL-12-P40: interleukin-12, subunit beta
IL-10: interleukin-10
IL-17A: interleukin-17, alpha
IL-1B: interleukin-1, beta
IL-1RA: interleukin-1 receptor antagonist
IL-6: interleukin-6
IL-8: interleukin-8
TNFa: tumor necrosis factor, alpha
VEGF: vascular endothelial growth factor
ATIII: antithrombin III
D-Dimer: D-dimer
HSP70: heat shock protein 70
PAI-1: plasminogen activator inhibitor-1
Pentraxin: pentraxin
RAGE: receptor for advanced glycation endproducts
TNFRI: tumor necrosis factor receptor 1
Sfas-L: soluble FAS-ligand
