# Supplementary material for: Tumor necrosis factor receptor 1 (TNFRI) for ventilator-associated pneumonia diagnosis by cytokine multiplex analysis
Source: Intensive Care Med Exp. 2015 Sep 16;3:26. doi: 10.1186/s40635-015-0062-1 (PMC4572048; doi:10.1186/s40635-015-0062-1)

**Electronic Supplement Material**

# Tumor necrosis factor receptor-1 (TNFR1) for ventilator associated pneumonia diagnosis by cytokine multiplex analysis

I Martin Loeches^1,2^ , L.D. Bos^3^, P Povoa^4-5^, P Ramirez^6^, M.J. Schultz^3^, A Torres^7^, A Artigas^2^

**Supplemental tables**

**Table S1:** Differences in biomarker concentration between VAP and no VAP on the day of VAP diagnosis

| Biomarker | No VAP N= 19 | | VAP N= 24 | | P-value |
| --- | --- | --- | --- | --- | --- |
|  | Median | 25-75th percentile | Median | 25-75th percentile |  |
| GCSF | 102.37 | [41.46-179.47] | 491.59 | [213.11-1101.1] | 0.0013 |
| IL-12-P40 | 3.2 | [3.2-3.6] | 3.2 | [3.2-5.59] | 0.724 |
| IL-10 | 10.7 | [6.47-19.16] | 32.75 | [16.93-80.47] | 0.0115 |
| IL-17A | 3.2 | [3.1-5.57] | 3.89 | [1.38-7.9] | 0.6856 |
| IL-1B | 3.2 | [2.29-3.2] | 3.2 | [3.2-3.2] | 0.3378 |
| IL-1RA | 35.7 | [13.86-85.96] | 42.4 | [20.17-137.82] | 0.3258 |
| IL-6 | 16.23 | [9.68-33.42] | 95.04 | [43.58-178.62] | 0.0019 |
| IL-8 | 20.55 | [8.86-25.96] | 63.11 | [38.65-142.95] | <0.001 |
| TNFa | 9.77 | [6.94-18.44] | 23.72 | [15.78-31.45] | 0.0172 |
| VEGF | 131.26 | [43.97-301.57] | 190.8 | [135.18-359.04] | 0.3844 |
| ATIII | 256639.3 | [141084.52-312845.06] | 222198.9 | [165360.15-413528.67] | 0.8085 |
| D-Dimer | 2490.92 | [1713.53-3200.78] | 2426.01 | [1759.56-3015.41] | 0.9655 |
| HSP70 | 151003.2 | [68442.9-383331.23] | 229060.2 | [141959.93-471544.36] | 0.2576 |
| PAI1 | 39067.78 | [11840.57-59673.11] | 39205.05 | [21512.69-76597.82] | 0.3556 |
| Pentraxin | 7.24 | [4.82-11.71] | 13.46 | [5.08-41.49] | 0.2341 |
| RAGE | 12.88 | [12.2-31.54] | 32.09 | [12.2-112.61] | 0.1093 |
| TNFR1 | 1418.31 | [941.41-1637.37] | 3958.84 | [2256.92-5476.49] | <0.001 |
| Sfas-L | 16.93 | [6.22-38.78] | 20.17 | [10.92-30.9] | 0.9424 |

**Table S2**: Differences in biomarker concentration between VAP and no VAP three days before VAP diagnosis

| Biomarker | No VAP N= 19 | | VAP N= 24 | | P-value |
| --- | --- | --- | --- | --- | --- |
|  | Median | 25-75th percentile | Median | 25-75th percentile |  |
| GCSF | 227.18 | [177.3-579.54] | 368.66 | [111.42-1052.07] | 0.9711 |
| IL-12-P40 | 3.2 | [3.2-8.19] | 3.2 | [3.2-3.2] | 0.0342 |
| IL-10 | 13.01 | [7.23-70.42] | 45.4 | [11.97-123.05] | 0.334 |
| IL-17A | 3.2 | [1.42-3.2] | 3.2 | [1.57-4.08] | 0.6801 |
| IL-1B | 3.2 | [3.2-3.2] | 3.2 | [3.2-3.2] | 0.2094 |
| IL-1RA | 55.95 | [10.85-65.89] | 33.46 | [14.08-89.09] | 0.893 |
| IL-6 | 35.51 | [25.84-130.38] | 122.13 | [26.13-450.06] | 0.2014 |
| IL-8 | 20.8 | [16.5-64.21] | 59.57 | [31.8-130.97] | 0.0258 |
| TNFa | 12.75 | [6.86-18.43] | 15.28 | [9.33-22.84] | 0.3501 |
| VEGF | 135.05 | [28.27-188.37] | 157.84 | [70.18-303.97] | 0.3371 |
| ATIII | 199544.55 | [163968.65-367566.29] | 208245.33 | [119996.16-598229.47] | 0.9903 |
| D-Dimer | 2275.11 | [1794.16-2691.87] | 2396.29 | [1838.15-3628.21] | 0.3629 |
| HSP70 | 105066.86 | [69186.56-336073.23] | 350882.07 | [150848.13-631146.3] | 0.0543 |
| PAI1 | 60697.28 | [37045.45-110872.04] | 136577.1 | [60488.85-283156.37] | 0.0171 |
| Pentraxin | 14.46 | [9.52-24.76] | 8.77 | [4.83-42.48] | 0.9903 |
| RAGE | 51.34 | [17.86-157.42] | 47.21 | [13.79-85] | 0.5646 |
| TNFR1 | 1660.74 | [1007.1-2032.64] | 3285.9 | [1852.29-8416.16] | 0.0031 |
| Sfas-L | 40.03 | [25.31-57.63] | 33.99 | [23.69-53.03] | 0.893 |

**Table S3**: Differences in biomarker slope between VAP and no VAP

| Biomarker change in concentration | No VAP N= 19 | | VAP N= 24 | | P-value |
| --- | --- | --- | --- | --- | --- |
|  | Median | 25-75th percentile | Median | 25-75th percentile |  |
| GCSF | -123.2 | (-409.5--74.7) | -16.8 | (-598.7-995.2) | 0.338 |
| IL-12-P40 | 0 | (-2-0) | 0 | (0-0.9) | 0.046 |
| IL-10 | -1.2 | (-50.6-1.3) | 1.7 | (-91.6-36.4) | 0.323 |
| IL-17A | 0 | (-0.1-1.1) | 0.9 | (-1.8-4.2) | 0.515 |
| IL-1B | 0 | (0-0) | 0 | (0-0.2) | 0.553 |
| IL-1RA | 6.7 | (-30.8-28.5) | 3.1 | (-11.5-82.7) | 0.544 |
| IL-6 | -14.5 | (-78.1--6.5) | -0.9 | (-246.6-78.8) | 0.558 |
| IL-8 | -6.7 | (-43.5-0.6) | 7.1 | (-41.1-37.3) | 0.223 |
| TNFa | 1.6 | (-3.2-4.7) | 3.3 | (-2.1-9.8) | 0.246 |
| VEGF | 28.3 | (-34.5-115.9) | 53.1 | (-12.2-181.7) | 0.654 |
| ATIII | -16102.3 | (-44493.1-27541.6) | 16589.4 | (-107931.8-93772.2) | 0.743 |
| D-Dimer | 135.2 | (-498.5-604.9) | 236.7 | (-669.9-870.1) | 0.743 |
| HSP70 | -17243 | (-96414.9--23.5) | -132684.4 | (-358643.8-26258.4) | 0.212 |
| PAI1 | -26746.4 | (-84412.7-1105.9) | -83425 | (-166604.3--33258.3) | 0.022 |
| Pentraxin | -5.5 | (-13.8-2.1) | -0.8 | (-14.7-12.3) | 0.399 |
| RAGE | -32.3 | (-72-4.1) | -14 | (-47.6-8.5) | 0.573 |
| TNFR1 | 129.2 | (-442.5-265.8) | 356.5 | (-3137.1-1027.2) | 0.521 |
| Sfas-L | -7.6 | (-22.8-0.9) | -10.3 | (-22.7-3.6) | 0.874 |

**Table S4**: Receiver operating characteristics per biomarker

| Biomarker | ROC | ROC 2.5% | ROC 97.5% | Cut-off | Sens | Spec | LR+ | LR- |
| --- | --- | --- | --- | --- | --- | --- | --- | --- |
| GCSF | 0.8 | 0.66 | 0.94 | 208.71 | 0.75 | 0.8 | 3.75 | 0.31 |
| IL-12-P40 | 0.53 | 0.36 | 0.7 | 3.28 | 0.33 | 0.73 | 1.25 | 0.91 |
| IL-10 | 0.74 | 0.58 | 0.91 | 13.98 | 0.79 | 0.73 | 2.97 | 0.28 |
| IL-17A | 0.54 | 0.35 | 0.73 | 3.22 | 0.58 | 0.6 | 1.46 | 0.69 |
| IL-1B | 0.58 | 0.42 | 0.74 | 2.29 | 0.88 | 0.27 | 1.19 | 0.47 |
| IL-1RA | 0.6 | 0.41 | 0.78 | 36.13 | 0.58 | 0.53 | 1.25 | 0.78 |
| IL-6 | 0.8 | 0.66 | 0.94 | 41.96 | 0.75 | 0.8 | 3.75 | 0.31 |
| IL-8 | 0.88 | 0.76 | 0.99 | 27.78 | 0.83 | 0.87 | 6.25 | 0.19 |
| TNFa | 0.73 | 0.56 | 0.9 | 20.41 | 0.67 | 0.8 | 3.33 | 0.42 |
| VEGF | 0.58 | 0.39 | 0.78 | 170.99 | 0.67 | 0.6 | 1.67 | 0.56 |
| ATIII | 0.48 | 0.29 | 0.66 | 248427.8 | 0.62 | 0.53 | 1.34 | 0.7 |
| D-Dimer | 0.51 | 0.31 | 0.7 | 2991.95 | 0.75 | 0.4 | 1.25 | 0.62 |
| HSP70 | 0.61 | 0.42 | 0.8 | 112446.69 | 0.88 | 0.47 | 1.64 | 0.27 |
| PAI1 | 0.59 | 0.39 | 0.79 | 16957.77 | 0.83 | 0.47 | 1.56 | 0.36 |
| Pentraxin | 0.62 | 0.44 | 0.8 | 12.43 | 0.54 | 0.8 | 2.71 | 0.57 |
| RAGE | 0.65 | 0.48 | 0.83 | 26.86 | 0.62 | 0.73 | 2.34 | 0.51 |
| TNFR1 | 0.91 | 0.82 | 1.0 | 1949.09 | 0.83 | 0.87 | 6.25 | 0.19 |
| Sfas-L | 0.51 | 0.31 | 0.71 | 17.34 | 0.58 | 0.53 | 1.25 | 0.78 |

**Table S5: Receiver operating characteristics per biomarker slope**

| Biomarker slope | ROC | ROC 2.5% | ROC 97.5% | Cut-off | Sens | Spec | LR+ | LR- |
| --- | --- | --- | --- | --- | --- | --- | --- | --- |
| GCSF | 0.59 | 0.41 | 0.78 | -27.66 | 0.54 | 0.87 | 4.06 | 0.53 |
| IL-12-P40 | 0.68 | 0.52 | 0.84 | 0.07 | 0.38 | 0.87 | 2.81 | 0.72 |
| IL-10 | 0.6 | 0.42 | 0.78 | 1.97 | 0.5 | 0.8 | 2.5 | 0.62 |
| IL-17A | 0.56 | 0.38 | 0.75 | 1.84 | 0.5 | 0.8 | 2.5 | 0.62 |
| IL-1B | 0.55 | 0.39 | 0.71 | 0.91 | 0.25 | 0.87 | 1.88 | 0.87 |
| IL-1RA | 0.44 | 0.25 | 0.63 | -1.96 | 0.46 | 0.67 | 1.37 | 0.81 |
| IL-6 | 0.56 | 0.37 | 0.74 | -3.52 | 0.54 | 0.8 | 2.71 | 0.57 |
| IL-8 | 0.62 | 0.44 | 0.8 | 3.78 | 0.54 | 0.87 | 4.06 | 0.53 |
| TNFa | 0.61 | 0.44 | 0.79 | 2.91 | 0.54 | 0.67 | 1.62 | 0.69 |
| VEGF | 0.54 | 0.35 | 0.74 | 46.53 | 0.54 | 0.6 | 1.35 | 0.76 |
| ATIII | 0.53 | 0.35 | 0.72 | 7783.07 | 0.54 | 0.73 | 2.03 | 0.63 |
| D-Dimer | 0.53 | 0.35 | 0.72 | 242.25 | 0.5 | 0.67 | 1.5 | 0.75 |
| HSP70 | 0.62 | 0.44 | 0.8 | -114770.24 | 0.54 | 0.8 | 2.71 | 0.57 |
| PAI1 | 0.72 | 0.55 | 0.89 | -34124.84 | 0.75 | 0.6 | 1.88 | 0.42 |
| Pentraxin | 0.58 | 0.4 | 0.77 | -1.47 | 0.54 | 0.67 | 1.62 | 0.69 |
| RAGE | 0.56 | 0.37 | 0.75 | -30.21 | 0.71 | 0.53 | 1.52 | 0.55 |
| TNFR1 | 0.56 | 0.37 | 0.76 | 408.03 | 0.5 | 1 | Inf | 0.5 |
| Sfas-L | 0.52 | 0.33 | 0.7 | -18.63 | 0.42 | 0.73 | 1.56 | 0.8 |

**Supplemental figures**

**Figure S1:** ROC analysis of biomarkers (A) at the day of diagnosis and (B) three days before.


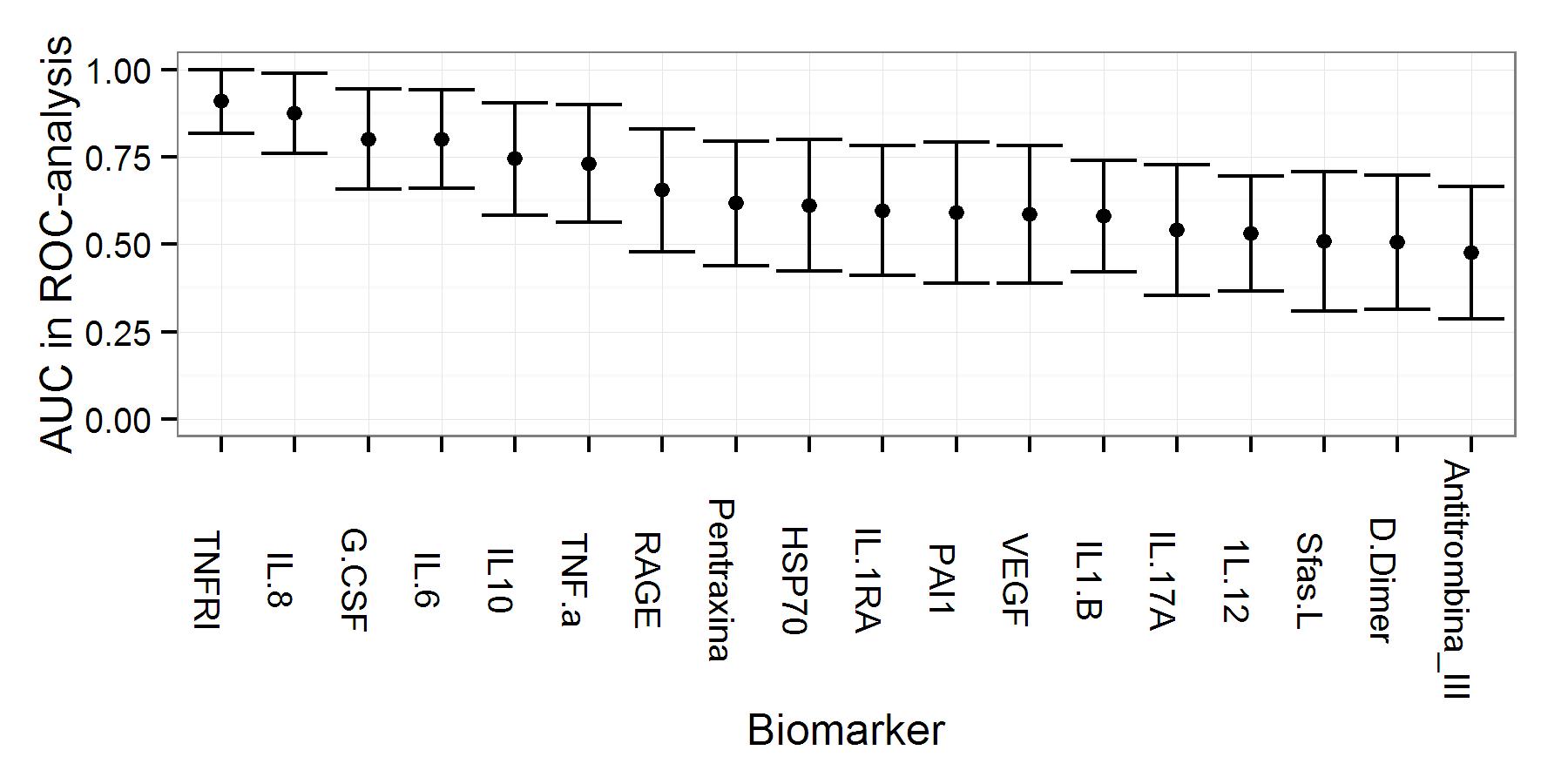


**
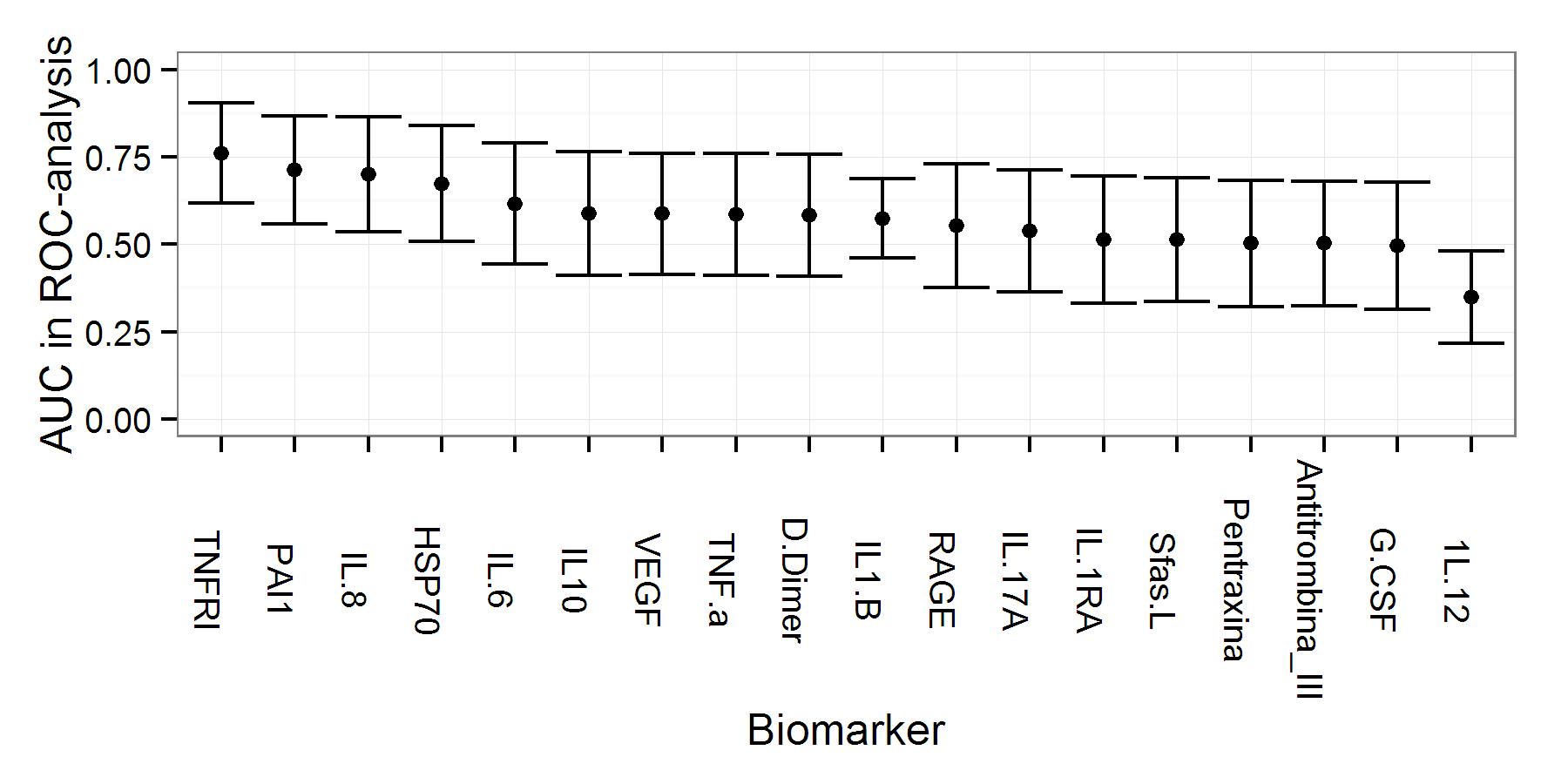
**

**Figure S2:** ROC of best biomarker model at the day of diagnosis


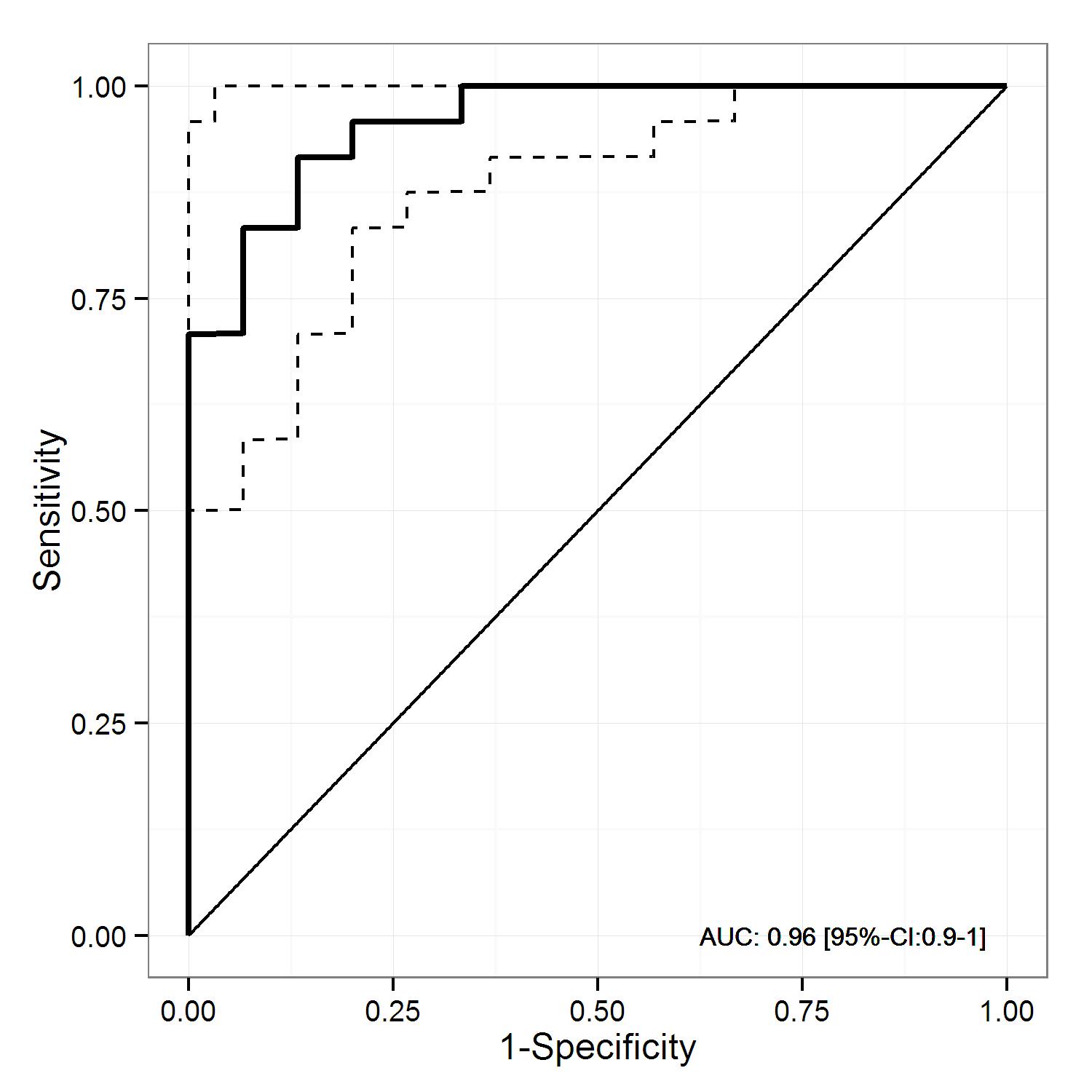


**Figure S3:** ROC analysis of biomarkers three days before the diagnosis


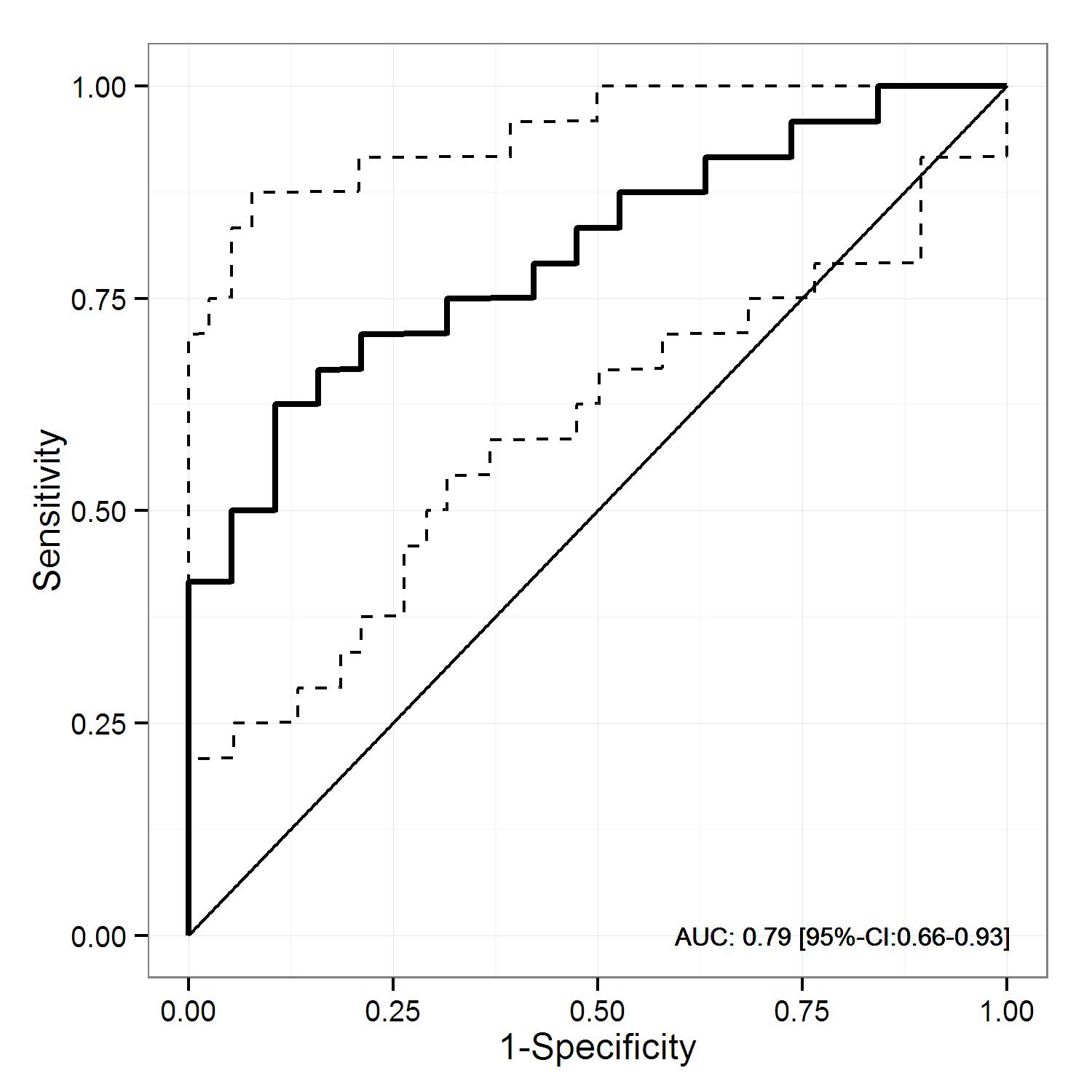


**Figure S5:** ROC of best biomarker slope model


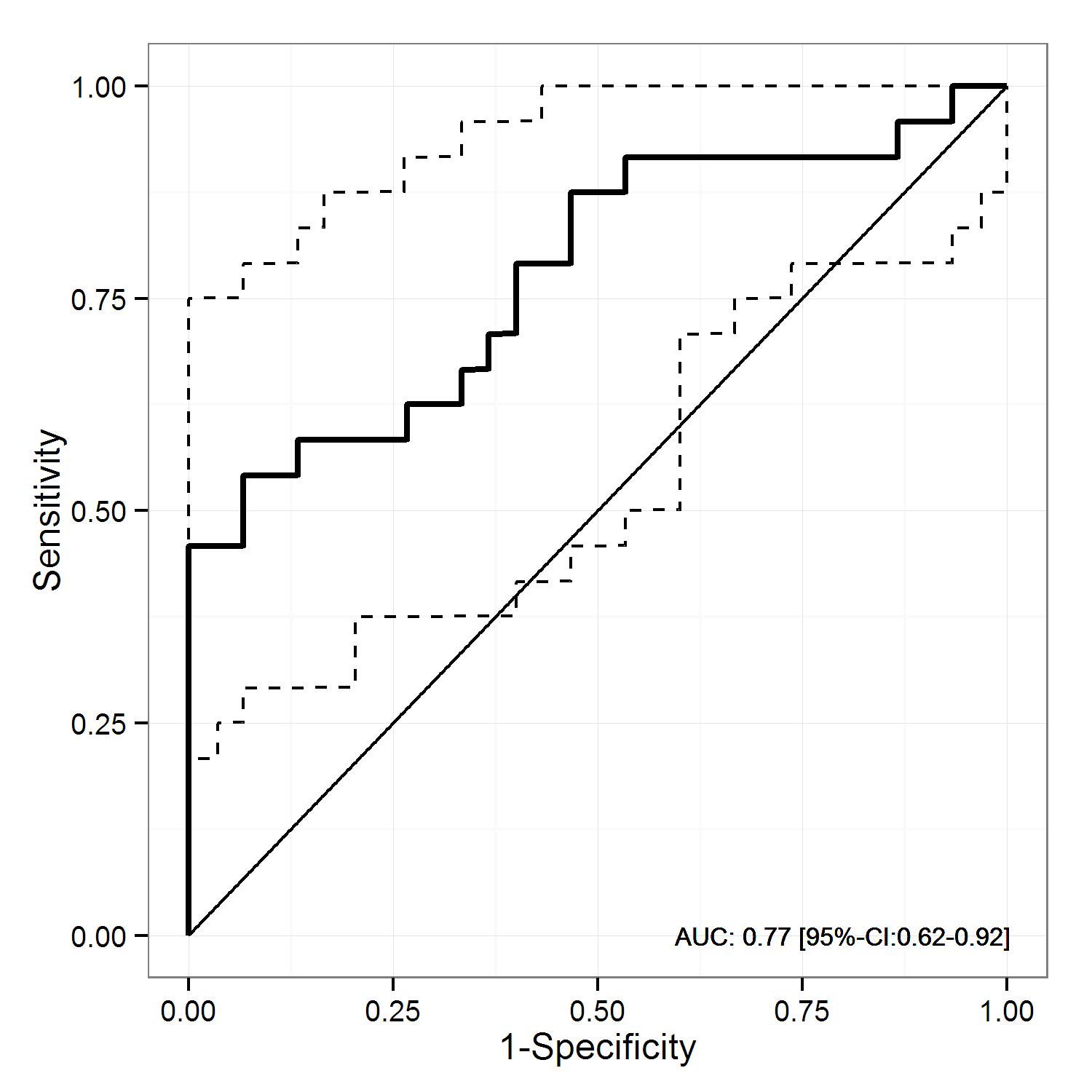

Supplement: Additional file 1: — Electronic supplement material. (DOC 702 kb) [file 40635_2015_62_MOESM1_ESM.doc]
